# Pre-Pregnancy Body Mass Index and Risk of Macrosomia and Large for Gestational Age Births with Gestational Diabetes Mellitus as a Mediator: A Prospective Cohort Study in Central China

**DOI:** 10.3390/nu14051072

**Published:** 2022-03-03

**Authors:** Xinli Song, Jing Shu, Senmao Zhang, Letao Chen, Jingyi Diao, Jinqi Li, Yihuan Li, Jianhui Wei, Yiping Liu, Mengting Sun, Tingting Wang, Jiabi Qin

**Affiliations:** 1 Department of Epidemiology and Health Statistics, Xiangya School of Public Health, Central South University, 110 Xiangya Road, Changsha 410078, China; xinlisong@foxmail.com (X.S.); sj1234511@163.com (J.S.); zsmhncs@163.com (S.Z.); chenletao93@163.com (L.C.); djy1996@csu.edu.cn (J.D.); ljq0280@126.com (J.L.); yihuan0911@163.com (Y.L.); weijihi@163.com (J.W.); 18843113354@163.com (Y.L.); sunmtbeloved@163.com (M.S.); 2 National Health Committee Key Laboratory of Birth Defect for Research and Prevention, Hunan Provincial Maternal and Child Health Care Hospital, 52 Xiangchun Road, Changsha 410028, China; 3 Guangdong Cardiovascular Institute, Guangdong Provincial People’s Hospital, Guangdong Academy of Medical Sciences, 106 Zhongshan Second Road, Guangzhou 510080, China; 4 Hunan Provincial Key Laboratory of Clinical Epidemiology, 110 Xiangya Road, Changsha 410078, China

**Keywords:** pre-pregnancy body mass index, macrosomia, large for gestational age, gestational diabetes mellitus, mediation analyses

## Abstract

This study aimed to examine the risk of macrosomia and large for gestational age (LGA) births in relation to maternal pre-pregnancy body mass index (BMI) status mediated through gestational diabetes mellitus (GDM). This prospective study included 34,104 singleton pregnancies at 8–14 weeks of gestation. The interesting outcomes were macrosomia (≥4000 g) and LGA (≥90th percentile). Mediation analyses were conducted using log-binomial regression adjusted for age, education, parity, fetal sex, and gestational weight gain. The proportion mediated was estimated based on the risk difference scale, and the *E*-value was utilized to assess potential confounders. Overall, 15.9% of women had GDM, and there were 4.0% macrosomia and 9.9% LGA births. The proportion mediated by GDM on macrosomia was up to 40% among obese women, and the estimate of the total effect was 6.18 (95% CI: 5.26–7.26), of the natural direct effect was 4.10 (95% CI: 3.35–4.99), and of the natural indirect effect was 1.51 (95% CI: 1.31–1.76). Likewise, among overweight women, the proportion mediated by GDM on macrosomia was up to 40%. Furthermore, consistent findings were evident for the outcome of LGA births. Pre-pregnancy overweight/obesity increased the risk of macrosomia and LGA births independently and partly mediated by GDM.

## 1. Introduction

Gestational diabetes mellitus (GDM) is defined as any degree of glucose intolerance with onset or first recognition during pregnancy [1]. The prevalence of GDM has been dramatically rising around the world, with around 15% in China [2], posing a great threat to maternal and neonatal health. About 15–45% of babies born to mothers with GDM are macrosomic, which is a three-fold higher risk than for normoglycemic controls [3]. Lifestyle interventions of GDM decrease the risk of macrosomic newborns, albeit not all evidence supports this [4]. According to the American College of Obstetrics and Gynecology, macrosomia and large for gestational age (LGA) are two terms that are applied to excessive fetal growth [5]. LGA generally indicates a birth weight equal to or more than the 90th percentile, respective of a given gestational age, and macrosomia implies growth beyond 4000 g, regardless of the gestational age [5]. Macrosomia and LGA newborns, accounting for approximately 9% of singleton live birth infants in China [6], not only present a quandary in terms of diagnosis and delivery options to avoid trauma (e.g., shoulder dystocia, nerve injuries, and birth asphyxia) [7,8,9], but are also related to long-term health outcomes such as obesity, type 2 diabetes, and cardiovascular problems in both the mother and child later in life [10,11]. The pathophysiology of macrosomic fetuses can be partly explained by the Pedersen’s hypothesis, and it is suggested that fetal overgrowth is related to increased glucose consumption, fetal hyperinsulinemia, and subsequent enlarged fetal adipose tissue [12]. Maternal pre-pregnancy obesity and GDM have been identified as the most major risk factors for both macrosomia and LGA infants, with maternal obesity being a stronger determinant than GDM [6,13,14]. Furthermore, it is well established that pre-pregnancy overweight/obesity is associated with an increased risk of GDM [15,16]. GDM is a well-known result of maternal overweight/obesity, as well as a contributing factor for increased fetal size, thereby implying GDM might have a mediation effect in this causal pathway. Therefore, we hypothesized that GDM could act as a mediator in the relationship between pre-pregnancy body mass index (BMI) and macrosomic infants in Chinese populations, and that the estimates of the mediation effect might differ between macrosomia and LGA births due to the distinctions in their definitions.

Hitherto, Kondracki and colleagues [17] reported a cross-sectional study in American populations that explored the role of GDM as a mediator in the association of maternal pre-pregnancy BMI on LGA infants, but none focused on macrosomia; thus, this work represents both the first report and replication efforts in a Chinese cohort to corroborate and extend the observations of Kondracki and colleagues. Considering the persistently high prevalence of GDM and fetal overgrowth in China, as well as the adverse effects of these conditions, understanding whether GDM acts as a mediator in the causal pathway of maternal pre-pregnancy BMI on macrosomia and LGA births would help point to chances to enhance perinatal outcomes. A novelty in this study was the use of the *E*-value approach recently introduced by VanderWeele and Ding [18,19], instead of sensitivity analysis, to assess the potential impact of unmeasured confounding. The *E*-value was defined as the minimum strength of association that an unmeasured confounder would require to explain away a given association, based on measured covariates [20]. With this context in mind, the present study aimed to investigate the risk of macrosomia and LGA births in relation to maternal BMI at the first antenatal care visit mediated through GDM, using a prospective cohort of pregnant women from Changsha, China.

## 2. Materials and Methods

### 2.1. The Study Population

**Figure A1:**
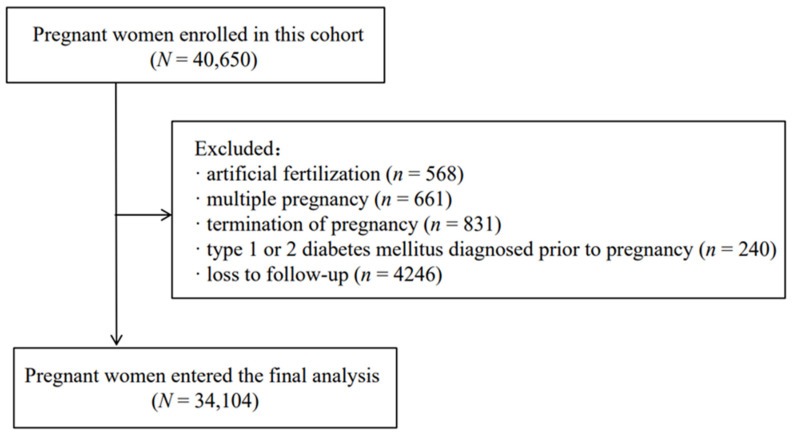
Flow chart showing the process of participant’s recruitment.

This prospective cohort study was conducted in Hunan Province, Central China, at the Hunan Provincial Maternal and Child Health Care Hospital, a provincial health center for mothers and children. From 13 March 2013 to 31 December 2019, pregnant women (≥18 years) who underwent their first prenatal visit at 8–14 gestational weeks and intended to continuously receive prenatal care throughout pregnancy at the study hospital were approached and invited to participate in this cohort. Gestational weeks were determined using ultrasonography if menstruation was irregular, or were approximated using the previous menstrual period data [21]. Additionally, pregnant women who met any of the following criteria were excluded: (1) artificial fertilization, (2) multiple pregnancies, (3) termination of pregnancy by artificial abortion or induced labor because of accidental pregnancy or ectopic pregnancy, or (4) type 1 or 2 diabetes mellitus diagnosed prior to pregnancy. Finally, a total of 40,650 pregnant women who met the inclusion criteria were recruited into this present cohort during their first prenatal care. After taking into account the exclusion criteria and loss to follow-up, 34,104 eligible pregnant women were included in the final analysis. The reasons for not including other pregnant women were as follows: (i) artificial fertilization (*n* = 568; 1.4%), (ii) multiple pregnancy (*n* = 661; 1.6%), (iii) termination of pregnancy (*n* = 831; 2.0%), (iv) type 1 or 2 diabetes mellitus diagnosed prior to pregnancy (*n* = 240; 0.6%), or (v) loss to follow-up (*n* = 4246; 10.5%) (Figure A1).

This study was conducted in line with the principles of the Declaration of Helsinki. Approval was granted by the Ethics Committee for Clinical Research of Xiangya School of Public Health of Central South University (no. XYGW-2018-36). Informed consent was acquired from all participants before data collection. Furthermore, we registered this study in the Chinese Clinical Trial Registry Center (registration number: ChiCTR1800016635; date of registration: 14 June 2018).

### 2.2. Information Collection

We gathered data using study-specific questionnaires and the database of the Electronic Maternal and Child Health Information System. From registration to delivery, this electronic system recorded clinical and biochemical samples from mothers and infants. After being recruited, participants who gave informed consent underwent a face-to-face interview by specially trained investigators who employed a self-designed questionnaire to gather information on maternal characteristics and pre-pregnancy BMI. Data on maternal GDM, maternal gestational week, and infant characteristics (i.e., infant’s weight and sex) were obtained from medical records.

### 2.3. Outcome

Macrosomia implied a birth weight equal to or beyond 4000 g, irrespective of the gestational age [5]. LGA generally indicated a birth weight equal to or more than the gender specific 90th percentile, respective of a given gestational age [5].

### 2.4. Exposure

All participants were measured for their height and weight with light clothing and no shoes on. BMI was computed by dividing body weight in kilograms by body height in meters squared. According to Chinese adult’s criteria, BMI was classified into four categories: underweight (<18.5 kg/m^2^), normal weight (18.5–23.9 kg/m^2^), overweight (24.0–27.9 kg/m^2^), and obesity (≥28.0 kg/m^2^) [22].

### 2.5. Mediator

GDM is a known consequence of maternal obesity and a risk factor for fetal overgrowth, and was a potential mediator. Participants underwent a standard 75-g 2-h oral glucose tolerance test (OGTT) between 24 and 32 weeks. The OGTT was performed in the morning after the subjects had fasted for more than 8 h. Plasma glucose levels at fasting, 1-h, and 2-h were determined using an automated analyzer (Toshiba TBA-120FR, Tokyo, Japan) at the Central Laboratory of the Hunan Provincial Maternal and Child Health Care Hospital. GDM was diagnosed using the cut-points established by the International Association of Diabetes and Pregnancy Study Group: 5.1 mmol/L in fasting plasma glucose, 10.0 mmol/L in 1-h plasma glucose, or 8.5 mmol/L in 2-h plasma glucose [23].

### 2.6. Covariates

Covariates were known as potential confounders, related with the exposure, mediator, and outcome. The following is a list of the potential confounders, which were selected based on a review of the relevant literature [24,25,26,27,28,29]: maternal age at pregnancy onset (<25, 25–29, 30–34 or ≥35), educational attainment (high school or less, some college, or bachelor’s+), parity (primipara or multipara), infant sex (male or female), and gestational weight gain (<10, 10–20 or ≥20 kg).

### 2.7. Statistical Analyses

**Figure 1:**
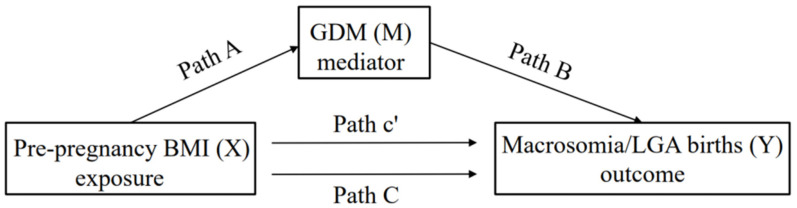
The total effect (Path C), direct effect (Path c’), and indirect effect (Path A and Path B) of the association among the exposure (X), outcome (Y), and mediator (M) are illustrated.

A distribution of maternal and newborn characteristics in this study sample of singleton births (*n* = 34,104) was initially described according to GDM, macrosomia and LGA newborns. The prevalence of GDM, macrosomia, and LGA newborns was estimated across maternal pre-pregnancy BMI categories, and their 95% confidence intervals (95% CIs) were calculated based on an approximation of the binomial distribution to the normal distribution where applying the central limit theorem was appropriate. Next, the mediation method based on the counterfactual framework for causal inference was used in this study [30,31]. In the mediation analysis, the total effect (TE) of a connection between an exposure and an outcome was separated via a mediator into the natural direct effect (NDE) and the natural indirect effect (NIE) (Figure 1). The NIE represented the effect of causal pathway, while all other mechanisms were represented by the NDE. In addition, Path A (mediator model) was used to estimate the impact of pre-pregnancy BMI upon GDM, and Path B (outcome model) was used to estimate the impact of GDM upon macrosomia/LGA newborns (Figure 1). Notably, the findings that GDM was strongly associated with both pre-pregnancy BMI and fetal macrosomia/LGA births were a necessary requirement for further mediation analysis. The estimates of the mediation effect were calculated using log-binomial (log-linear) regression models, and were adjusted for age, education, parity, infant sex (only for macrosomia), and gestational weight gain, and were reported as relative risk ratios (RRs) and their 95% CIs [31]. We also calculated the proportion mediated by GDM that contributed to the total effect on the risk difference scale [32,33]. The *E*-value approach was utilized in this study to assess the sensitivity to potential unmeasured confounding, and it was calculated using the estimates and their upper and lower limits of the 95% CIs [18,34]. All statistical analyses were performed in Statistical Analysis System (SAS), release 9.4 (SAS Institute Inc., Cary North Carolina, USA). A two-tailed *p* value < 0.05 was considered to indicate statistical significance.

## 3. Result

### 3.1. Characteristics of Participants

Overall, 15.9% (*n* = 5430) of pregnant women had GDM, and macrosomia and LGA births accounted for 4.0% (*n* = 1374) and 9.9% (*n* = 3359), respectively (Table 1). Most women had a normal pre-pregnancy BMI (70.2%), 14.4% and 12.7% were underweight and overweight, respectively, while the minority of women (2.7%) were in the obesity category. Additionally, the majority of women had a college degree (50.9%), and more than 70% were aged between 25 and 34, including 34.8% between 25 and 29 and 37.5% between 30 and 34.

### 3.2. Prevalence of GDM, Macrosomia and LGA Births across Maternal Pre-Pregnancy BMI Status

The prevalence of GDM varied by maternal pre-pregnancy BMI status (Table 2), ranging from 6.4% among underweight women to 22.3% among obese women. Likewise, the prevalence of macrosomia and LGA infants ranged from 2.5% to 14.5% and 9.1% to 30.7% among underweight and obese women, respectively.

### 3.3. Mediation Analysis

In the Path A models, overweight (aRR 1.60 (95% CI 1.48–1.74)) and obese (aRR 2.34 (95% CI 2.02–2.71)) women were associated with a higher risk of GDM, while underweight women (aRR 0.62 (95% CI 0.56–0.69)) were associated with a lower risk of GDM. In the Path B models, GDM was associated with both macrosomia (overweight women aRR 1.61 (95% CI 1.39–1.85), obese women aRR 1.62 (95% CI 1.39–1.88), and underweight women aRR 1.39 (95% CI 1.17–1.63), respectively) and LGA births (overweight women aRR 1.24 (95% CI 1.12–1.37), obese women aRR 1.37 (95% CI 1.23–1.52), and underweight women aRR 1.19 (95% CI 1.07–1.33), respectively) (Table 3). GDM was significantly associated with both pre-pregnancy BMI and fetal macrosomia/LGA risk, and thus a mediation analysis was performed to evaluate whether GDM mediated the relationship of pre-pregnancy BMI with fetal macrosomia/LGA.

The estimates of the TE, NDE, and NIE in association with each pre-pregnancy BMI category on macrosomia and LGA births were statistically significant, with less than 1 being in the underweight category and larger than 1 being in the overweight/obese category (Table 3). Compared with those who had normal pre-pregnancy BMI, overweight women (aRR_NDE_ 1.40 (95% CI 1.20–1.62) and aRR_TE_ 1.75 (95% CI 1.56–1.96)) and obese women (aRR_NDE_ 4.10 (95% CI 3.35–4.99) and aRR_TE_ 6.18 (95% CI 5.26–7.26)) were directly associated with a higher risk of fetal macrosomia, while underweight women were directly associated with a lower risk of macrosomia (aRR_NDE_ 0.66 (95% CI 0.54–0.79) and aRR_TE_ 0.56 (95% CI 0.49–0.64)). Additionally, there was an additional mediated effect due to GDM when mothers were overweight (aRR_NIE_ 1.25 (95% CI 1.16–1.36)), obese (aRR_NIE_ 1.51 (95% CI 1.31–1.76)), and underweight (aRR_NIE_ 0.86 (95% CI 0.78–0.93)). For the outcome of LGA infants, similar findings were evident when mothers were overweight (aRR_NDE_ 1.34 (95% CI 1.21–1.49) and aRR_TE_ 1.49 (95% CI 1.37–1.62)), obese (aRR_NDE_ 2.63 (95% CI 2.23–3.09) and aRR_TE_ 3.44 (95% CI 3.02–3.92)), and underweight (aRR_NDE_ 0.62 (95% CI 0.55–0.70) and aRR_TE_ 0.57 (95% CI 0.52–0.63)), again with evidence of an additional mediated effect due to GDM (overweight women aRR_NIE_ 1.11 (95% CI 1.05–1.17), obese women aRR_NIE_ 1.31 (95% CI 1.19–1.46), and underweight women aRR_NIE_ 0.92 (95% CI 0.87–0.97)) (Table 3).

We also calculated the proportion mediated by GDM. For the outcome of macrosomia births, the estimated proportion mediated by GDM in overweight, obese, and underweight women was 46.7%, 40.3%, and 21.4%, respectively. When it came to the outcome of LGA births, the estimated proportion mediated was 30.2%, 33.3%, and 11.5% among the overweight, obese, and underweight category, respectively.

### 3.4. Assessment of Unmeasured Confounding

The *E*-values (Table 4) were larger than the estimates of the NDE and NIE, particularly for obese and macrosomia births (the aRR_NDE_ = 7.67, lower 95% CI = 6.16; the aRR_NIE_ = 2.39, lower 95% CI = 1.95). The NDE was estimated to be 4.10 with an *E*-value of 7.67, indicating that NDE would be explained by unmeasured confounding related to both, maternal obesity, and macrosomia by an odds ratio of 7.67-fold or greater. Similarly, NIE was estimated to be 1.51 with a corresponding *E*-value of 2.39, suggesting that the effect could be explained away by unmeasured confounding by an odds ratio of 2.39-fold, above any measured confounding, while weaker confounding could not. In summary, our findings show that any unobserved confounder could be adequate to fully explain away these effect estimates and to move the CIs to null, while a weak confounder could not do so.

## 4. Discussion

We aimed to understand the role of GDM as a mediator in association with maternal pre-pregnancy BMI on fetal macrosomia and LGA births in the offspring. By evaluating the prevalence and mediation effects of GDM, this study drew attention to the disease burden of overweight and obese pregnant women with GDM. An estimated 15.9% of women developed GDM during pregnancy, and more than 15% were overweight or obese, in line with other recent reports in China [16]. Growing evidence supports that maternal overweight/obesity and GDM are the most major determinants for macrosomia and LGA infants [6,13,14], and this study also observed these significantly independent associations. Furthermore, our results suggested that GDM might act as a potential mediator. The proportion mediated, which answered the causal question of how much of the total effect of the association was explained by GDM, was the highest among overweight pregnant women, reaching up to 40% for macrosomia and 30% for LGA births. Kondracki and colleagues [17] analyzed a cross-sectional database based on American populations (*n* = 3,801,534), and revealed a potential role of GDM as a mediator in LGA newborns, which was consistent with our findings. However, most likely due to the discrepancies in race and the condition of GDM and overweight/obesity among woman of childbearing age between countries, the highest mediated proportion (up to 16%) observed by Kondracki et al. [17] was relatively lower than that observed in this study. Babu and colleagues [35] recruited a cohort of 1120 women of all BMI ranges from Bangalore, India, and revealed a mediator effect of GDM between maternal obesity and neonatal adiposity. Given the generalized increase in body fat of the macrosomic fetuses [36], the observations of Babu and colleagues also provided evidence for our findings. Although the mechanism through which maternal overweight/obesity and glycemia, alone or in combination, influenced the intrauterine microenvironment and fetal development remained unknown, the association appeared biologically plausible. According to the Pedersen hypothesis [12], fetus macrosomia born to mothers with GDM reflected the impact of fetal hyperglycemia and subsequent hyperinsulinaemia caused by maternal hyperglycemia, implying that the growth-promoting actions of both glucose and insulin were responsible for fetal overgrowth and fat mass accretion.

This study screened for GDM at the recommended 24–28 weeks of gestation, according to Chinese guidelines, and observed a significant association between GDM and macrosomic newborns, which was consistent with prior studies on GDM and increased fetal growth in late pregnancy (i.e., after 24 weeks of gestation) [37,38,39]. When GDM occurred in the setting of overweight/obesity, even if therapies maintained glucose within the target range, the fetus could overgrow owing to an excess of nutrition being shunted [40]. However, several studies have reported that early screening (before 20 weeks of gestation) and adequate therapy of GDM can prevent against the start of fetal overgrowth, thus suggesting that screening for GDM should begin before the recommended 24–28 weeks of gestation. Li and colleagues [41] explored the timing of fetal growth alteration in relation to maternal glycemic status during gestation in a large, multiracial, prospective cohort study, and they discovered that the association between GDM and larger fetal size emerged initially at 20 weeks of gestation and became statistically significant at week 28, despite adherence to standard clinical treatment for GDM. In addition, regardless of subsequent GDM diagnosis, rising tertiles of glucose levels at weeks 10–14 were found to be significantly related to larger fetal growth in late pregnancy, according to the observations of Li and colleagues. Subsequently, Chiefari et al. [42] compared the fetal size between women with GDM diagnosed at 16–18 weeks of gestation and treated promptly, and women with GDM diagnosed at 24–28 weeks of gestation owing to a failure to cooperate with early screening suggestions, and they found the women with an earlier GDM diagnosis had a smaller fetal size.

Our study also observed that maternal pre-pregnancy overweight/obesity significantly increased the risk of GDM and macrosomic newborns, and prior literature has confirmed this topic in different races [15]. Additionally, maternal overweight/obesity and GDM have been shown to raise the offspring’s propensity to obesity, poor glucose control, and GDM, causing a vicious cycle that leads to a cumulative risk in the following generation [43]. Lifestyle interventions targeting healthy nutrition and physical exercise well before pregnancy may help to lessen overweight and obesity, and adherence to a healthy lifestyle prior to pregnancy is related with a lower GDM risk [44]. The American College of Obstetricians and Gynecologists recommended that women’s BMI be calculated at their first prenatal visit, and that proper weight gain, diet, and exercise be reviewed at both the first visit and at regular intervals during gestation [5]. So far, safe, easily applicable, and effective interventions to apply these guidelines throughout pregnancy are still required to prevent GDM and the consequent short- and long-term health outcomes for both the mother and the child.

Our results represent the potentially considerable effects of maternal overweight or obesity on fetal growth via GDM in the glucose/insulin pathway. However, it is apparent that GDM was not the sole contributor and there might be several undetected or undiscovered pathways though which additional factors act as potential shared mechanisms. It has been reported that fetuses of overweight women are consistently larger than average for all ultrasonography biometry parameters [45], implying maternal overweight/obesity is directly associated with an early and significant effect on fetal growth [37,45]. Furthermore, obesity is a complicated condition characterized by multiple altered metabolic pathways [46]. Metabolic factors such as circulating triglycerides [47], leptin [48], and adiponectin [49] are associated with fetal birth weight, although the mechanisms remain unclear.

Our study has several strengths. First, a major strength is the large sample size and prospective data collection. Second, applying the counterfactual or potential outcomes approach to mediation analysis is very advantageous, because the mediator varies naturally with the exposure on the outcome to offer insight into pathway-specific effect estimates. Last, the *E*-value approach is utilized based on measured variables to provide an assessment of the sensitivity/robustness to potential unmeasured confounding. Our study is not without limitations. First, all participants were from a single city region and our findings need replications in other pregnant women populations in China. Second, the blood glucose profiles of the offspring were not tested, which may be helpful to explain the proposed association between maternal GDM and fetal overgrowth. Finally, the various forms of intervention for GDM, including proper weight gain, healthy diet, physical exercise, regular glucose monitoring, and medication therapy could also affect fetal growth. Because there were a lack of data for these interventions of pregnant women with GDM in this study, this was a preliminary investigation on this topic, and future studies should evaluate the mediation effects of GDM in this causal framework stratified by diet, exercise, or medication interventions.

## 5. Conclusions

Our findings suggest pre-pregnancy overweight/obesity increased the risks of macrosomia and LGA newborns independently and considerably mediated via GDM. With the persistently high prevalence of GDM and macrosomic births in China, there is an urgent need for effective interventions aiming at preventing, early screening, and adequate treatment of GDM to decrease the consequent short- and long-term health outcomes for both the mother and the child. In addition, concerns about GDM and fetal overgrowth should be included in weight-control interventions targeting overweight or obese women throughout the whole pregnancy.

## Figures and Tables

**Table 1:** Distribution of maternal and infant characteristics according to GDM, macrosomia, and LGA newborns in the study sample of singleton births (*n* = 34,104).

Maternal and Infant Characteristics	Total Births *n* (%)	GDM *n* (%)	Macrosomia *n* (%)	LGA *n* (%)
	34,104	5430 (15.9%)	1374 (4.0%)	3359 (9.9%)
Pre-pregnancy BMI (kg/m^2^)				
Underweight (<18.5)	4920 (14.4)	448 (8.3)	122 (8.9)	315 (9.4)
Normal (18.5–23.9)	23,925 (70.2)	3696 (68.1)	888 (64.6)	2300 (68.5)
Overweight (24.0–27.9)	4334 (12.7)	1002 (18.5)	230 (16.7)	538 (16.0)
Obese (≥28.0)	925 (2.7)	284 (5.2)	134 (9.8)	206 (6.1)
Age at pregnancy onset				
<25	1769 (5.2)	126 (2.3)	78 (5.7)	151 (4.5)
25–29	11,873 (34.8)	1412 (26.0)	472 (34.4)	1266 (37.7)
30–34	12,803 (37.5)	2168 (39.9)	532 (38.7)	1246 (37.1)
≥35	7659 (22.5)	1724 (31.7)	292 (21.3)	696 (20.7)
Education				
High school or less	12,242 (35.9)	1865 (34.3)	470 (34.2)	1109 (33.0)
Some college	17,351 (50.9)	2877 (53.0)	722 (52.5)	1820 (54.2)
Bachelor’s or higher	4511 (13.2)	688 (12.7)	182 (13.2)	430 (12.8)
Parity				
Primipara	16,446 (48.2)	2486 (45.8)	653 (47.5)	1707 (50.8)
Multipara	17,658 (51.8)	2944 (54.2)	721 (52.5)	1652 (49.2)
Infant sex				
Male	17,953 (52.6)	2743 (50.5)	883 (64.3)	2139 (63.7)
Female	16,151 (47.4)	2687 (49.5)	491 (35.7)	1220 (36.3)

BMI = body mass index; GDM = gestational diabetes mellitus; LGA = large for gestational age.

**Table 2:** Prevalence (% and 95% CI) of GDM, macrosomia, and LGA newborns according to maternal pre-pregnancy BMI.

Pre-Pregnancy BMI (kg/m^2^)	GDM % (95% CI)	Macrosomia % (95% CI)	LGA % (95% CI)
Total	9.8 (9.5–10.2)	4.0 (3.8–4.2)	15.9 (15.5–16.3)
Underweight (<18.5)	6.4 (5.7–7.1)	2.5 (2.0–2.9)	9.1 (8.3–9.9)
Normal (18.5–23.9)	9.6 (9.2–10.0)	3.7 (3.5–4.0)	15.4 (15.0–15.9)
Overweight (24.0–27.9)	12.4 (11.4–13.4)	5.3 (4.6–6.0)	23.1 (21.9–24.4)
Obese (≥28.0)	22.3 (19.6–25.0)	14.5 (12.2–16.8)	30.7 (27.7–33.7)

BMI = body mass index; GDM = gestational diabetes mellitus; LGA = large for gestational age; 95% CI = 95% confidence interval.

**Table 3:** The estimated total effect, natural direct effect, natural indirect effect, and Path A and Path B for the association of pre-pregnancy BMI on macrosomia and LGA births with GDM as a mediator using a log-binomial model.

Pre-Pregnancy BMI (kg/m^2^)	Total Effect	Natural Direct Effect	Natural Indirect Effect	Path A	Path B	Proportion Mediated
	aRR_TE_ (95% CI)	aRR_NDE_ (95% CI)	aRR_NIE_ (95% CI)	aRR (95% CI)	aRR (95% CI)	%
Adjusted risk ratio of fetal macrosomia						
Normal (18.5–23.9)	1.00 (Ref.)	1.00 (Ref.)	1.00 (Ref.)	1.00 (Ref.)	1.00 (Ref.)	-
Underweight (<18.5)	0.56 (0.49–0.64)	0.66 (0.54–0.79)	0.86 (0.78–0.93)	0.62 (0.56–0.69)	1.39 (1.17–1.63)	21.4
Overweight (24.0–27.9)	1.75 (1.56–1.96)	1.40 (1.20–1.62)	1.25 (1.16–1.36)	1.60 (1.48–1.74)	1.61 (1.39–1.85)	46.7
Obese (≥28.0)	6.18 (5.26–7.26)	4.10 (3.35–4.99)	1.51 (1.31–1.76)	2.34 (2.02–2.71)	1.62 (1.39–1.88)	40.3
Adjusted risk ratio of LGA						
Normal (18.5–23.9)	1.00 (Ref.)	1.00 (Ref.)	1.00 (Ref.)	1.00 (Ref.)	1.00 (Ref.)	-
Underweight (<18.5)	0.57 (0.52–0.63)	0.62 (0.55–0.70)	0.92 (0.87–0.97)	0.62 (0.56–0.69)	1.19 (1.07–1.33)	11.5
Overweight (24.0–27.9)	1.49 (1.37–1.62)	1.34 (1.21–1.49)	1.11 (1.05–1.17)	1.60 (1.48–1.74)	1.24 (1.12–1.37)	30.2
Obese (≥28.0)	3.44 (3.02–3.92)	2.63 (2.23–3.09)	1.31 (1.19–1.46)	2.34 (2.02–2.71)	1.37 (1.23–1.52)	33.3

Note: Adjusted for age at pregnancy onset, education, parity and infant sex; Path A (mediator model): the effect of pre-pregnancy BMI on GDM; Path B (outcome model): the effect of GDM on macrosomia/LGA births. BMI = body mass index; GDM = gestational diabetes mellitus; LGA = large for gestational age; aRR = adjusted risk ratio; 95% CI = 95% confidence interval.

**Table 4:** Mediational *E*-value analysis for macrosomia and LGA births.

Pre-Pregnancy BMI (kg/m^2^)	Natural Direct Effect		Natural Indirect Effect	
	Adjusted Risk Ratio	Upper/Lower Confidence Limit	Adjusted Risk Ratio	Upper/Lower Confidence Limit
Adjusted risk ratio of fetal macrosomia				
Underweight (<18.5)	2.40	Upper 1.85	1.60	Upper 1.36
Overweight (24.0–27.9)	2.15	Lower 1.69	1.81	Lower 1.59
Obese (≥28.0)	7.67	Lower 6.16	2.39	Lower 1.95
Adjusted risk ratio of LGA				
Underweight (<18.5)	2.61	Upper 2.21	1.39	Upper 1.21
Overweight (24.0–27.9)	2.01	Lower 1.71	1.46	Lower 1.28
Obese (≥28.0)	4.70	Lower 3.89	1.95	Lower 1.67

BMI = body mass index; GDM = gestational diabetes mellitus; LGA = large for gestational age.

## Data Availability

The data presented in this study are available upon request from the corresponding author.
